# *In Situ* Characterization of the Local Work Function along Individual Free Standing Nanowire by Electrostatic Deflection

**DOI:** 10.1038/srep21270

**Published:** 2016-02-17

**Authors:** Yicong Chen, Chengchun Zhao, Feng Huang, Runze Zhan, Shaozhi Deng, Ningsheng Xu, Jun Chen

**Affiliations:** 1 State Key Lab of Optoelectronic Materials and Technologies, Sun Yat-sen University, Guangdong 510275, People’s Republic of China; 2 Guangdong Province Key Lab of Display Material and Technology, Sun Yat-sen University, Guangdong 510275, People’s Republic of China; 3 School of Physics and Engineering, Sun Yat-sen University, Guangdong 510275, People’s Republic of China; 4 School of Microelectronics, Sun Yat-sen University, Guangdong 510275, People’s Republic of China

## Abstract

*In situ* characterization of the work function of quasi one dimensional nanomaterials is essential for exploring their applications. Here we proposed to use the electrostatic deflection induced by work function difference between nanoprobe and nanowire for *in situ* measuring the local work function along a free standing nanowire. The physical mechanism for the measurement was discussed in details and a parabolic relationship between the deflection and the potential difference was derived. As a demonstration, measurement of the local work functions on the tip and the sidewall of a ZnO nanowire with Au catalyst at its end and a LaB_6_ nanowire have been achieved with good accuracy.

Quasi one dimensional (Q1D) nanomaterials such as nanowires have been regarded as the building blocks in the development of nanoscale electronics and optoelectronics^1^,^2^,^3^,^4^,^5^,^6^,^7^, however, our knowledge in this field is still limited. The characterization of Q1D nanomaterials is essential for the understanding of their physical nature and application. To be specific, junctions which can be formed within Q1D nanomaterials themselves or by contacting with other materials are the basis for most of the electronic and optoelectronic devices. Their characteristics are related to the contact barrier and the contact potential which are both mainly determined by the work function. Thus, an *in situ* characterization of the local work function of Q1D nanomaterials is of great significance for studying its junction or the contact issues in the device applications.

So far, Kelvin probe force microscope (KPFM) is the only technique to realize this characterization in the nanoscale, and the contact potential difference (CPD) along individual homogeneous or heterogeneous nanowires have been widely studied by it^8^,^9^,^10^. Although this technique has a very high spatial resolution and measuring accuracy, it also has critical requirements on the roughness and purity of the sample surface. Its measurement procedure is complicated, especially for the as-grown free standing Q1D nanomaterials which are generally applied in electronic and optoelectronic devices. Usually, Q1D nanomaterials are dispersed on a flat substrate by a chemical solvent which can be destructive to the sample surface. Besides, the inevitable interaction between the sample and the substrate, especially of the nanomaterials, has a negative impact on the characterization of the intrinsic properties of Q1D nanomaterials. *In situ* measurement of the work function of individual free standing Q1D nanomaterials was firstly carried out by Wang’s group^11^,^12^. Based on the electromechanical resonance of Q1D nanomaterials, their proposed *in situ* TEM technique has already been applied to measure the work functions of individual free standing CNT^11^, ZnO nanobelt and the attached carbon particle on the tip of the nanobelt^12^. However, this technique is restricted to the measurement on the tip of Q1D nanomaterials.

In this work, a measurement technique based on the electrostatic deflections of Q1D nanomaterials has been proposed, which could fulfill the task of *in situ* characterization of work function distribution along free standing Q1D nanostructures. As an example, the work function of the tip and the sidewall of an individual free standing ZnO nanowire with Au catalyst at its end have been measured to be 4.52~4.79e V and 4.93~5.06e V *via* a nanoprobe coated with Au layer. While that of a LaB_6_ nanowire have been measured to be 3.76e V and 3.74e V *via* a bare tungsten probe. The *in situ* technique can be extended to other free standing Q1D nanostructure devices such as field emitters. These results are important for the understanding of the nature of Q1D nanomaterials and the development of their applications.

## Results

### Theoretical basis

**Figure 1:**
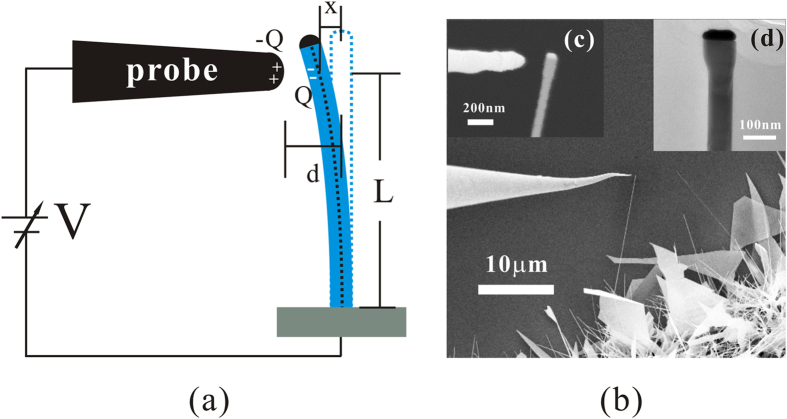
Principle of the method. (**a**) Schematic diagram of the measurement. When the probe moves approach different positions of the nanowire with a distance of d, charge Q can be induced at the corresponding position due to their work function difference. As a result, a bending deflection x of the nanowire can be caused due to their electrostatic attractive force. By adjusting the external voltage V, their potential difference can be eliminated and there will be no electrostatic deflection. (**b**) SEM image of the measurement where the insets are (**c**) the enlarged SEM image and (**d**) the TEM image of the nanowire.

The detail of our measuring method is illustrated in Fig. 1(a). Considering the root of a free standing nanowire is electrically connected to the nanoprobe *via* the substrate. When the tip of the nanoprobe moves closely to a local position of the nanowire, a static charge Q can be induced there due to their different surface potential, which results from their different work function (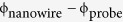) even though the external voltage V is zero. According to the Gauss law, the quantity of Q is proportional to the electric field near surface E_s_, 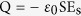, where ε_0_ is the permittivity of vacuum and S is the area of the induced charge on the nanowire which is related to the geometry and distance between nanowire and nanoprobe. Modeling a nanowire as a one-dimensional object, the electric field between the nanoprobe and the nanowire near surface E_s_ can be described as


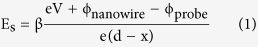


where β is the field enhancement factor, e is the charge of an electron, d is the distance between the tip of the nanoprobe and the central axis of the nanowire when there is no bending deflection and x is the bending displacement of the measuring point which have been symbolized in Fig. 1(a). The induced charge will cause an electrostatic attractive force F_1_ between the nanoprobe and the nanowire which can be described as


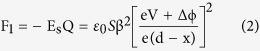


where 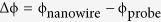. As a result, the nanowire will have a bending deflection x toward the nanoprobe.

In the equilibrium state, the electrostatic force F_1_ is equal to the force F_2_ required to overcome the elastic energy which is given by^13^


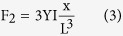


where Y is the elastic modulus, I is the momentum of inertia and L is the length between the measuring point and the root of the nanowire under the small angle deflection approximation. For the hexagonal cross section nanowire, 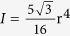, where r is the radius of the nanowire.

**Figure 2:**
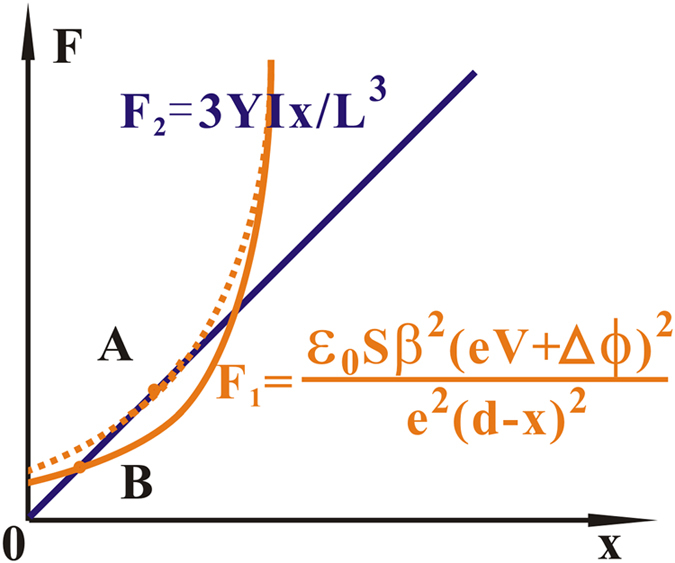
The relationship between the electrostatic force F_1_ and the force F_2_ required to overcome the elastic energy. F_1_ and F_2_ are represented by the orange and blue curves respectively. With a different value of 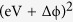, F_1_ can be intersecting (solid line) or tangent (dash line) with F_2_.

The relationship between F_1_ and F_2_ is represented in Fig. 2. When the distance d is fixed, equation (2) will be intersecting or tangent with equation (3) at point B or A under different value of 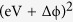. The significance of this result is that when F_1_ is intersecting with F_2_ at point B, the nanowire will bend to the x position of B because F_1_ will be smaller than F_2_ beyond this point. When F_1_ is tangent with F_2_ at point A, the nanowire will bend until it touches with the nanoprobe because F_1_ is always larger than or equal to F_2_. The x position of point B can be derived by the following equation


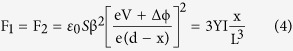


Assuming the induced charge area S and the field enhancement factor β can be treated as the constant, equation (4) can be simplified as


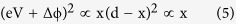


when x ≪ d, which indicates a parabola relationship between V and x. As 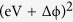 becomes zero, the bending deflection x will become zero since the electric force F_1_ is zero. This can be derived from either equation (4) or (5). Therefore, to measure the local work function of the nanowire is to find out the appropriate value of V which causes the minimum bending deflection of the nanowire. With a known work function of the nanoprobe 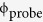, the local work function of nanowire can be obtained as 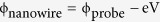.

**Figure 3:**
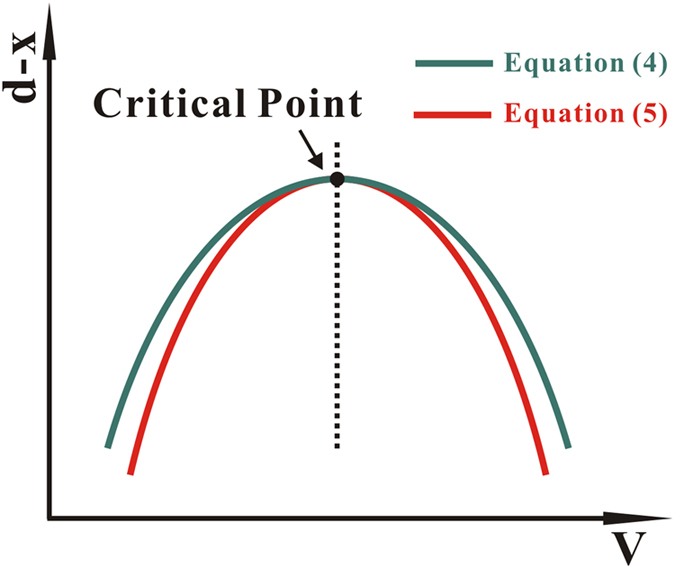
The relationship between equation (4,5). Equation (**4,5**) are represented as the green and red curves respectively. Although their converging form is different, they own the same vertex and symmetry axis.

Generally, since S(d − x) and β(d − x) are the function of d − x, equation (5) may have a deviation from equation (4) which can be seen in Fig. 3. However, they still own the same vertex and symmetry axis, which indicates that their critical points for x = 0 locate at the same position. Therefore, a parabola fitting can be used for obtaining the critical point in the measurement when x ≪ d.

Considering that the nanoprobe just induces charge at the region closed to it on the nanowire, it is possible to measure the local work function of different regions on the nanowire by moving the nanoprobe to approach there. And the spatial resolution is mainly relied on the curvature radius of the nanoprobe and the working distance.

### Measurement on a ZnO nanowire

Using this technique, the CPDs of the Au particle on the tip of an individual free standing ZnO nanowire and the sidewall region below it have been measured. High magnification SEM image [Fig. 1(c)] and TEM image [Fig. 1(d)] indicated that Au particle catalyst was located on the top of the nanowire. Experimentally, the tip was first moved closely to the nanowire in the direction perpendicular to the nanowire with an applied voltage of 1V which can be seen in Fig. 1(b). When the tip was moved to the critical position, the nanowire would bend and touch with the tip. After that, the tip was moved a little away from this critical position and the applied voltage swept from 1 V–−2 V until the nanowire bent and touched with the tip. The step of V is −0.1 V. The SEM images of the nanowire during each value of V were recorded.

**Figure 4:**
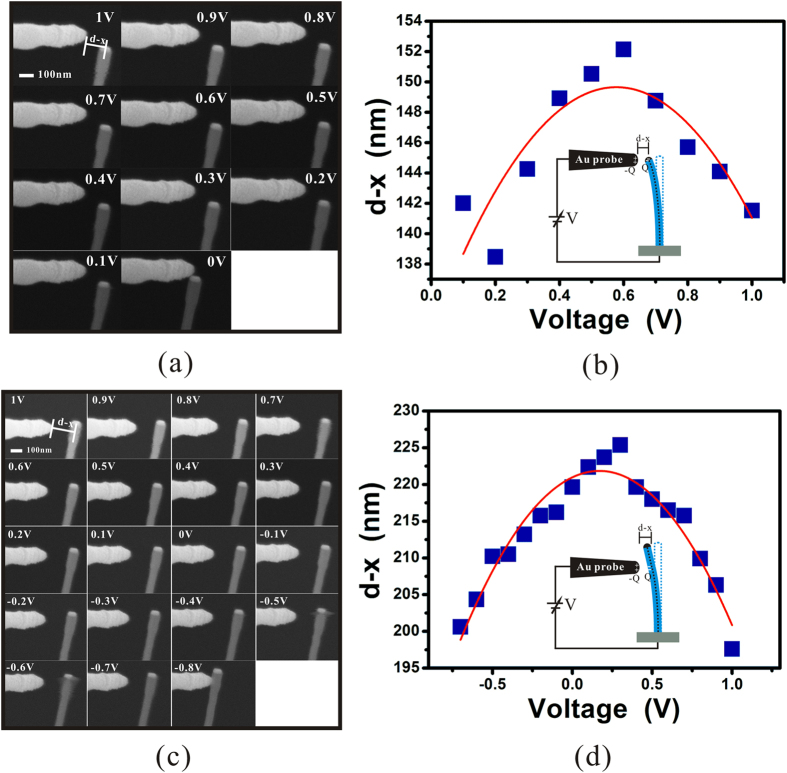
Measurement results on a ZnO nanowire. (**a**,**c**) are series of SEM images of the measurement on the Au particle and the sidewall region below it at a ZnO nanowire respectively with different external voltage V. (**b**,**d**) are their corresponding results of d – x versus the external voltage. The red curves are the results fitting by a parabola function, which show that their CPDs are 0.58 V and 0.17 V respectively when comparing to the Au-coated probe. The insets are the corresponding schematic diagrams of the measurements.

Figure 4 a, c are the typical SEM images of the measurement on the Au particle of a ZnO nanowire and the sidewall region below it respectively with different external voltage using a probe coated with Au. Fig. 4(b,d) are the corresponding plots of the distance between probe and nanowire d – x as a function of voltage V. Since d − x is in the range of 150–200 nm and x is in the range of 10–20 nm, equation (5) is validated. Therefore, a parabola fitting has been done which is represented as the red curves in Fig. 4(b,d). By extracting the vertex of the fitting results, one can obtain their CPDs as 0.58 V and 0.17 V respectively. Considering that the work function of Au is 5.1 eV, the work function of the Au particle on the top of the ZnO nanowire and the sidewall region below it are 4.52e V and 4.93e V, respectively.

It should be noted that the effect of electron beam should be considered here since the measurement was performed while the electron beam was exposed on the sample. According to some previous works^14^,^15^,^16^, electron beam induced deposition is significant in a vacuum pressure ranging from 10^−3^ mbar to 10^−8^ mbar. Considering that the measurement in this work is carried out in an ultra-high vacuum system with a base pressure of 10^−10^ mbar, the effect of electron beam induced deposition is neglected here. Since ZnO is a semiconductor, other effects of the electron beam, such as charge accumulation or the potential drop on the sample induced by the electron beam have been considered in this work. To our knowledge, charge accumulation and potential drop on the sample is proportional to the beam current. Therefore, the measurements have been done on 4 nanowires under different electron beam currents to see whether these effects will arise on the nanowire. The results are listed in Table 1. No obviously difference is found among the results. To ensure the accuracy of the method, the measurement has also been done by using a bare tungsten probe, the results of which are also similar considering that the work function of W is 4.5e V which can be seen in the Supplementary Fig. S1 online.

### Measurement on a LaB_6_ nanowire

**Figure 5:**
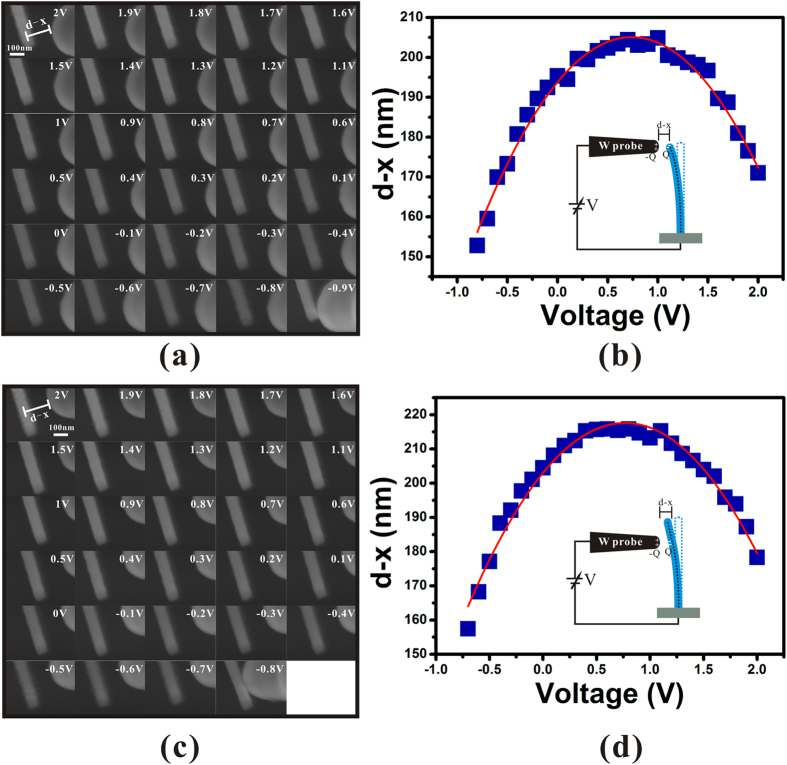
Measurement result on a LaB_6_ nanowire. (**a**,**c**) are series of SEM images of the measurement on the tip and the sidewall of a LaB_6_ nanowire respectively with different external voltage. (**b**,**d**) are the corresponding results of d − x versus V. The red curves are the results fitting by a parabola function, which show that their CPDs are 0.74 V and 0.76 V respectively when comparing to a tungsten probe. The insets are the corresponding schematic diagrams of the measurements.

To demonstrate the universality of this method, the CPDs of the tip and the sidewall of a LaB_6_ nanowire have also been measured. Considering the work function of the bulk LaB_6_ is 2.6–3.0 eV, the sweep voltage was started from 2 V on with a bare tungsten probe. The results are represented in Fig. 5. By extracting the vertex of their fitting results, their CPDs can be obtained as 0.74 V and 0.76 V respectively when comparing to a tungsten probe. Considering that the work function of tungsten is 4.5 eV, the work function of the tip and the sidewall of the LaB_6_ nanowire are both around 3.75 eV.

## Discussion

The work function of around 4.5 eV for the Au particle on the tip of ZnO nanowire is attributed to its composition of Au-Zn alloy. The synthesis mechanism of our sample can be described by the vapor-liquid-solid (VLS) mechanism. Generally, in the VLS model, the Zn vapor impinges on the Au particles and forms alloy droplets. When the droplets become supersaturated, crystalline ZnO nanowires are formed. This process continues as long as the temperature is held and the supply of Zn and O_2_ is sufficient. During the cooling process, the alloy droplet can solidify to Au or Au-Zn depending on the atmosphere and the temperature in the tube furnace^17^,^18^,^19^. In our case, Au-Zn alloy is preferred to be solidified on the tip of the nanowire. Considering that the work function of Zn is 4.33 eV, the mol ratio between Au and Zn in the Au-Zn alloy particle can be estimated roughly to be about 1:3 by using the mixing law of the work function of alloy (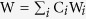, where C_*i*_ and W_*i*_ are the mol ratio and the work function of the *i*th specie of the material), which has also been reported before in a low temperature synthesis method^19^. To confirm the existent of Zn, energy-dispersive X-ray spectroscopy (EDX) measurement has also been done on the droplet, the result of which can be seen in the supplementary Fig. S2 online. The signals of Au and Zn with an atomic ratio of about 1:4 have been detected from the droplet, which suggests the result of our work function measurement is reasonable.

The work function of around 4.9 eV for the ZnO nanowire may be resulted from its higher carrier concentration comparing to the bulk ZnO. Generally, due to its small size in two dimensions, the surface states and defects have an important role on the properties of Q1D nanomaterials. As a result, the carrier concentration ranging from 10^19^ cm^−3^ to 10^20^ cm^−3^ have been reported for the undoped ZnO nanowire^20^,^21^, which is several orders higher than that of the bulk ZnO. For the N-type semiconductor, such as ZnO, a higher carrier concentration will increase the Fermi level and result in a lower work function.

Since ZnO is a piezoelectric material, its piezoelectric potential caused by the deflection was also considered here. According to the early theoretical works^22^,^23^, the piezoelectric potential is proportional to the lateral displacement of the nanowire and inversely proportional to the cube of its length-to-diameter ratio. Besides, it also related to the doping concentration. For example, Gao *et al.*^23^ calculated the piezoelectric potential of ZnO nanowire upon deflection. In their calculation, a lateral force of 80 nN applied on the top of a ZnO nanowire with length of 600 nm, diameter of 50 nm and doping concentration of 10^17^ cm^−3^ can cause a piezoelectric potential of −0.3 V at the compressed side. Considering the ZnO nanowire in this study is in a diameter of 100 nm and a length of more than 30 μm, and its maximum deflection is about 30 nm, one can estimate the corresponding piezoelectric potential is about −4 μV by using the parameters in the reference^23^. Furthermore, the resistance of our nanowire is in the order of MΩ as obtained from electrical measurement, which should have a doping concentration of larger than 10^17^ cm^−3^. This may also reduce the piezoelectric potential^23^. Therefore, it is believed that the influence of the piezoelectric potential can be neglected here.

The relatively high work function of around 3.75 eV for the LaB_6_ nanowire might be related to its amorphous layer on the surface, which has been observed in HRTEM. In early study, the amorphous layer on the LaB_6_ nanowire is believed to be composed of boron (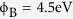), which results in a higher surface work function^24^. However, more experiments are needed to draw a conclusion.

In summary, an *in situ* technique based on the electrostatic deflection of Q1D nanomaterials has been proposed to measure its work function distribution. As an example, the work functions of the tip and the sidewall of a ZnO nanowire with Au catalyst at its end and a LaB_6_ nanowire have been measured by using this technique. For ZnO nanowire, the work function of the Au catalyst on its apex is around 4.5 eV which is attributed to its composition of Au-Zn alloy, while that of the sidewall is around 4.9  which arise from its higher carrier concentration. For LaB_6_ nanowire, its work function of 3.75  is larger than that of the bulk LaB_6_, which might be related to its surface amorphous layer. This work is important for the characterization of the intrinsic properties of Q1D nanomaterial, which can lead to a better understanding of their physical nature.

## Methods

### Material preparation

ZnO nanowires were grown by a CVD method in a horizontal tube furnace. Briefly, a 5-nm-thick Au film was first deposited on the Si substrate by electron beam evaporation. Then, an annealing process at 800 °C was done in the N_2_ atmosphere for 10 min to form Au particles. After that, the source (1:1 mass ratio of ZnO/C powders) and substrates were held at 910 °C and 825 °C, respectively, with a flow of 1sccm O_2_ and 200 sccm Ar under a pressure of 4000 Pa for 10–20 min.

### Material characterization

The structure of the ZnO nanowire and LaB_6_ nanowire were characterized by transmission electron microscopy (FEI Titan G2 60–300). The EDX analysis of the droplet of a ZnO nanowire was done using an Oxford Instruments X-MAX^N^ 20 silicon drift detector in a Zeiss SUPRA 60 SEM system.

### Measurements

The measurement was carried out in the nanoprobe system (Omicron Nanoscience) which is equipped with a SEM system and 4 STM probes. The base pressure of the ultrahigh vacuum chamber was 10^−10^ mbar. All the STM probes are manipulated by the piezo motor. The tungsten tips were made by a chemical etched process which have been coated with Au by magnetron sputtering deposition. The voltage on the tip was provided by a sourcemeter (Keithley 2450).

## Additional Information

**How to cite this article**: Chen, Y. *et al.* *In Situ* Characterization of the Local Work Function along Individual Free Standing Nanowire by Electrostatic Deflection. *Sci. Rep.* **6**, 21270; doi: 10.1038/srep21270 (2016).

## Supplementary Material

Supplementary Information

## Figures and Tables

**Table 1:** CPDs of the Au particle and the sidewall of the ZnO nanowires under different beam current of SEM using an Au-coated probe.

No.	CPD_Au particle_ (V)	CPD_sidewall_ (V)	Beam current
1	0.58	0.17	1nA
2	0.51	0.04	1nA
3	0.58	0.08	200pA
4	0.31	0.12	200pA
