# Supplementary material for: In Situ Characterization of the Local Work Function along Individual Free Standing Nanowire by Electrostatic Deflection
Source: Sci Rep. 2016 Feb 17;6:21270. doi: 10.1038/srep21270 (PMC4756696; doi:10.1038/srep21270)
Supplement: Supplementary Information [file srep21270-s1.pdf]

In Situ Characterization of the Local Work Function along Individual Free Standing Nanowire by Electrostatic Deflection

Yicong Chen, Chengchun Zhao, Feng Huang, Runze Zhan, Shaozhi Deng, Ningsheng Xu, Jun Chen

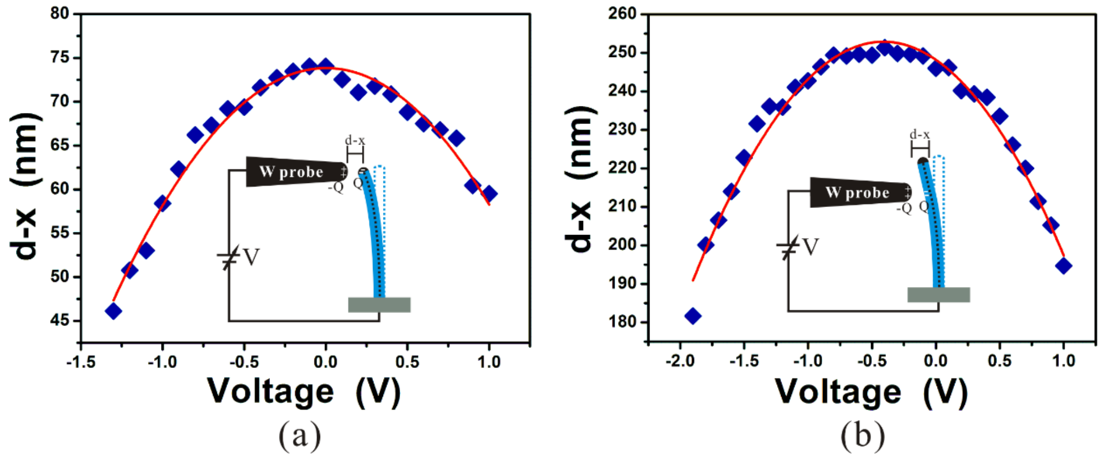

Figure S1. Measurement results using a bare tungsten probe. (a) and (b) are the plots of d-x versus external voltage V for the Au particle and the sidewall region below it at ZnO nanowire, where the red curves are the fitting result using a parabola function. From the fitting result, it is seen that their CPDs are 0V and -0.41V respectively. The insets are the corresponding schematic diagrams of the measurements. Considering that the work function of W is 4.5eV, the work function of the Au particle and the sidewall of ZnO nanowire are 4.5eV and 4.91eV respectively, which is in consistent with the results obtained by using the Au-coated probe.

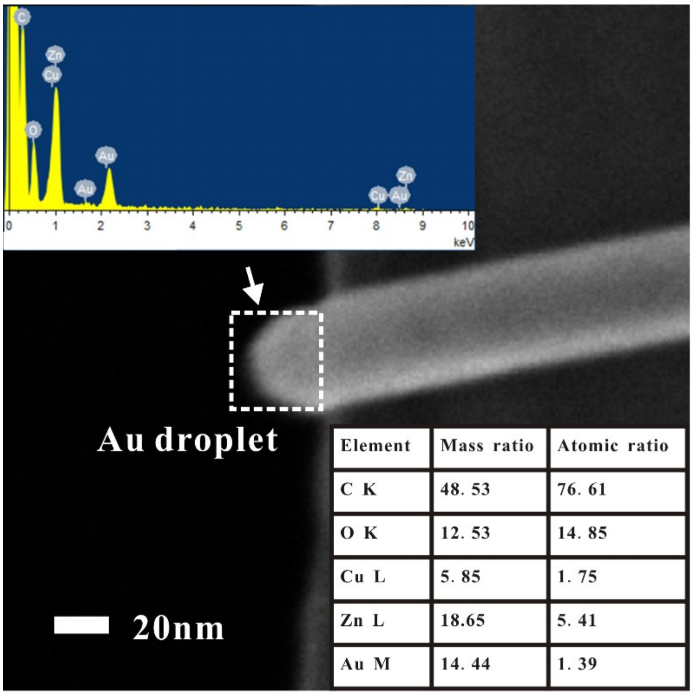

Element	Mass ratio	Atomic ratio
C K	48. 53	76. 61
O K	12. 53	14. 85
Cu L	5. 85	1. 75
Zn L	18.65	5. 41
Au M	14. 44	1. 39

Figure S2. EDX result of the Au droplet on a ZnO nanowire. The main picture is the SEM image of a ZnO nanowire. The rectangle region is the Au droplet, where the EDX signals detected from. Insets are the EDX spectrum and the corresponding mass ratio and atomic ratio among each element.
